# The association between oxytocin receptor gene polymorphism, childhood adversity and negative symptoms of schizophrenia

**DOI:** 10.1192/j.eurpsy.2025.1530

**Published:** 2025-08-26

**Authors:** T. Lezheiko, V. Golimbet, V. Mikhailova, M. Gabaeva

**Affiliations:** 1 Clinical Genetics Laboratory, Mental Health Research Center, Moscow, Russian Federation

## Abstract

**Introduction:**

Neurohormone oxytocin plays an important role in the pathogenesis of mental illness, and also moderates the relationship between stress factors, especially those acting in the early stages of development, and the development of mental disorders. Literature data indicate that environmental risk factors significantly increase the risk of schizophrenia and the severity of its clinical presentation.

**Objectives:**

To study the association between the oxytocin receptor (OXTR) rs468302 and rs7632287 polymorphisms and negative symptoms of schizophrenia, taking into childhood adversity (CA), i.e. events that could adversely affect the psychoemotional state and development of the child in the period up to 18 years. CA includes abuse in the family, alcohol or drug addiction in parents, mental disorders.

**Methods:**

The study included 592 patients with schizophrenia (items F20. according to ICD-10). Information about the presence of CA was obtained from case histories and patient interviews. Analysis of covariance was used for statistical data processing; in post-hoc pairwise comparison, Tukey’s test was used.

**Results:**

A significant effect of the interaction between CA and OXTR gene polymorphism rs7632287(G\A) on the severity of negative symptoms in patients with schizophrenia was revealed. For rs4686302 (C\T) polymorphism the association was found at the trend level. In patients without CA, polymorphisms did not have a significant effect.

**Conclusions:**

The OXTR rs468302 and rs7632287 polymorphisms, previously associated with phenotypes related to social behavior, may be associated with negative symptoms of schizophrenia, and the association is mediated by the presence of a history of psychotraumatic events acting at an early stage of development.

**Disclosure of Interest:**

None Declared

